# Legume Proteins and Peptides as Compounds in Nutraceuticals: A Structural Basis for Dietary Health Effects

**DOI:** 10.3390/nu14061188

**Published:** 2022-03-11

**Authors:** Marina Carbonaro, Alessandro Nucara

**Affiliations:** 1 Council for Agricultural Research and Economics (CREA), Research Centre for Food and Nutrition, Via Ardeatina 546, 00178 Rome, Italy; 2 Dipartimento di Fisica, Università Sapienza di Roma, Piazzale A. Moro 2, 00185 Rome, Italy; alessandro.nucara@roma1.infn.it

**Keywords:** legume proteins, nutraceuticals, structural properties, bioactive peptides, in vivo digestibility, health effects

## Abstract

In the current climate of food security, quality aspects of legume crops have primary market economic and health impact. Legume proteins and peptides have been discovered to have a role far beyond supplying amino acids for growth and maintenance of body tissues. Several proteins (enzymatic inhibitors, lectins, storage globulins) and peptides derived from them (lunasin, hydrophobic peptides) have shown anticarcinogenic, hypocholesterolemic, glucose-lowering, antioxidant, antimicrobial, and immunostimulant properties. Further understanding of how structural features of legume proteins affect in vivo digestion and production of bioactive sequences represents a key step in the valorization of nutraceutical potentiality of legume proteins and peptides derived from them. In this work, the relationship between structure and bioavailability of protein and peptides are reviewed and discussed.

## 1. Introduction

Legumes have a potential to add to the nutritional quality of foods and many options have been suggested for their inclusion in novel food preparation with improved nutritional and functional values.

Proteins represent one of the most concentrated nutrients in legumes, and they can be easily used as components in innovative human foods. In addition, legumes have higher protein content than cereals: therefore, they represent a primary source of amino acids for humans. Proteins extracted from legumes are an important font of proteins of plant origin, that can be consumed as an alternative to meat proteins [1].

Legumes, i.e., *Glycine max* (soybean) and *Phaseolus* species (beans), are grown in the tropical and subtropical areas of the world. It has been recognized that legumes have a primary role in the sustainability of agriculture, enhancing soil quality through nitrogen fixation.

Grain legumes (pulses) are included in the traditional diets of many countries. Indeed, dietary guidelines recommend increasing their consumption, especially in developed countries, due to their potential health benefits. They represent main sources of proteins and minerals (iron, zinc, calcium), while having a low amount of lipids, with the exception of soybean, peanut, and lupin (30–35%). Legume seeds contain low amounts of saturated fats. On the other hand, they are rich in carbohydrates (up to 60%), primarily starch, and in many vitamins (thiamine, niacin, biotin, riboflavin, folic acid). Moreover, legumes are a primary source of total dietary fiber (up to 37%) [1]. For this reason, they are a low-glycaemic food [2].

Epidemiological studies have highlighted a correlation between regular intake of legume seeds and maintenance of a good health status in humans. In meta-analyses of prospective observational studies, consumption of legumes has been related with a low risk of coronary heart disease [3,4].

The word “nutraceutical” was coined in the United States in 1989 to define “a food, or components of a food, that provides health benefits, including the prevention and treatment of diseases” [5].

Nutraceutical properties of legumes have been ascribed to non-nutrient compounds —isoflavones, alkaloids, phytates, saponins—and to a number of proteins such as enzymatic (protease and amylase) inhibitors, lectins, storage proteins—as well as to peptides [1]. It is worth reminding that most of these components have originally been considered as antinutrients, because of their adverse effects on nutrient metabolism [1].

## 2. Grain Legume Proteins

Legume proteins have attracted interest from the food industry. Indeed, they have a low cost, and good functional and nutritional attributes [6].

Proteins are accumulated in legume seeds during development inside protein bodies (storage vacuoles) and provide ammonia, carbon, and amino acids during seed development and to proteins. Proteins represent from about 20% in most legumes to 40% in soybean seeds [1]. Storage proteins are prevailing in legume seeds.

According to Osborne classification [7] storage proteins are divided into globulins, albumins, glutelins, and prolamins. Globulins are extracted in salt solutions, albumins are soluble in water, glutelins are soluble in acids or bases, and prolamins are soluble in ethanol [7]. Globulins are predominant in legume seeds, while prolamins and glutelins are prominent in cereals seeds [8]. Legumin, vicilin, and convicilin are the globulins present in legumes. Depending on the source, seeds usually present one or two predominant types of 81 storage proteins. Seed storage proteins also include enzymes, protease 82 inhibitors (trypsin, chymotrypsin, and amylase inhibitors), lectins, defense proteins, and others.

According to their sedimentation coefficient, globulins are divided into 7S and 11S oligomeric proteins. The 7S proteins are called vicilins, the 11S proteins are named legumins. The 7S/11S ratio is variable inside seeds and is dependent on the different legume species.

In *Phaseolus vulgaris* L., the predominant globulin is phaseolin, which accounts for 50% of total protein content, while in *Vicia faba* L. the most abundant globulin is vicilin (30% of total proteins). In *Lens culinaris* L. vicilin and legumin account for 72% and 11% of total proteins, respectively. In *Cicer arietinum* L. legumin and vicilin represent 66.5% and 12% of total protein content, respectively. In addition to the 7S and 11S proteins there are also the 2S proteins (monomeric proteins) [9].

Storage proteins are mostly oligomeric proteins. The 7S globulins are typically trimers (MW about 150 kDa), while the 11S proteins form hexamers (MW about 350–400 kDa), or higher association of subunits, such as the 15–18S globulins found in soybean globulins. From these oligomeric proteins, subunits can be released under dissociating conditions or upon thermal treatment. Reassociation of subunits can result in high-molecular weight aggregates with low susceptibility to digestion [10].

Other proteins present in legume seeds have antinutritional effects. The most important are protease inhibitors (Kunitz and Bowman–Birk inhibitors) and lectins, which have presented some adverse effects on animals and humans. However, the effects of these proteins are lost after processes preceding consumption (cooking, fermentation, germination, or dehulling) [11].

Globulins contain high content of aspartic acid and glutamic acids while albumins are rich in lysine and sulfur-containing amino acids [12]. Sulfur amino acids (methionine and cysteine) and tryptophan are limiting amino acids in legumes. On the other hand, they have a high amount of lysine, a property that makes them complementary to cereal proteins.

Because most of the albumin have higher sulfur amino acid content than globulin, strategies to increase this class of proteins in the seed is relevant. Among these, increasing the proportion of embryo axis to endosperm because the percentage of albumin is higher in the axis than in the endosperm [13]. Among globulins, legumins (11S proteins) have a higher sulfur amino acid content than 7S globulins. Therefore, increasing the legumin to vicilin ratio can result in an increase in sulfur amino acids in the seed. In the complex, plant proteins (legumes and cereals) have a lower nutritional quality than animal proteins.

Besides amino acid composition, studies focused on amino acid availability have pointed out that structural properties of plant proteins, making them resistant to gastrointestinal proteolysis, further lower the nutritional quality, because of limited bioavailability of essential amino acids [14]. Although trypsin inhibitors of many legumes are relatively high sulfur containing proteins, those of *Phaseolus vulgaris* L. and *Glycine max* L. (Bowman–Birk inhibitors) appear not to be readily digested in the rat gut [15].

Legume protein functional properties have been modified by application of several methods, such as thermal treatment, ultrasonication, and high pressure that affect protein structure [16]. In particular, high pressure caused a gradual unfolding of structure with improvement in solubility and emulsifying activity of kidney bean (*Phaseolus vulgaris* L.) isolate, due to the formation of soluble aggregates [17,18].

On the other hand, foaming properties of pea proteins were not improved by thermal treatment. An increase in hydrophobicity as a consequence of changes in structure was observed. Heat treatment induced loss of the oligomeric assembly, subunit denaturation with exposure of hydrophobic and sulfhydryl groups and reassociation into protein aggregates with modified surface properties and limited protein solubility [10,19,20].

Kidney bean and lentil protein isolates showed an improvement in solubility and emulsifying activity with the application of high pressure. However, after high pressure treatment, emulsion properties of kidney bean hydrolysate were impaired, as evidenced by secondary structure modifications (in particular, a shift in amide I and II of the infrared spectrum) [21].

When chickpea protein isolate was subjected to ultrasound treatment, an improvement in solubility, emulsifying, foaming and gel properties was observed [22].

Owing to their biocompatibility, film formation and functional (probiotics) properties, legume proteins have also been used in the encapsulation of several compounds [23]. They are effective for the preservation of probiotic and labile compounds during gastrointestinal digestion [24]. Folate, α-tocopherol, ascorbic acid, and phytase have been incapsulated in protein isolates from pea and chickpea, with 62–100% encapsulation efficiency and good release in the gastrointestinal tract [25,26].

## 3. Nutraceutical Properties of Legume Proteins

The health benefits of consumption of legume seeds have been reported in relation to many diseases, such as cancer, cardiovascular disease, the ageing process, immune response, diabetes, weight control, osteoporosis, digestive tract diseases, and mental health [27,28,29].

Small peptides, partially digested proteins and intact proteins from soybean, lupin, lentil, chickpea, pea, and the common bean, exert hormone-like activities [30,31]. Specific beneficial effects in humans include cardiovascular protection, anticancer activity, bone protection, control of weight, immune cell action, insulin sensitivity, control of inflammation and of type 2 diabetes mellitus [32,33,34,35]. Some examples of bioactive proteins and peptides derived from legume seeds are presented in Table 1.

Enzyme inhibitors present in legume seeds have been found to be active in the control of proteases, amylases, and glycosidases. These enzymes are involved in the mechanism of defense against exogenous attack (insects and microorganisms) [37].

Once inactivated, proteins known as antinutritional factors, that is protease inhibitors and lectins, have been reported to show health effects. Protease inhibitors are active towards inflammation and cancer [38]. Regular consumption of legumes has been shown to reduce the risk of several cancers, such as colon, prostate, gastric, and pancreatic cancer. Anticarcinogenic properties have been attributed to enzymatic inhibitors, especially Bowman–Birk inhibitors (BBI) [39,40]. Soybean, lentil, and pea BBI have been found to be active in the prevention and suppression of colon, liver, lung, prostate and mammalian cancers induced by chemical and physical agents, soybean BBI being particularly effective [41].

Control of protease activity by protease inhibitors may be responsible for their anticancer power.

α-Amylase inhibitors have shown antidiabetic activity and, therefore, potential applications in the control of obesity. Similarly, α-glucosidase inhibitors have been proposed in the treatment of diabetes. Delay of digestion and absorption of carbohydrates helps in the control of postprandial hyperglycemia in the diabetic patient.

Lectins are blood grouping substances, immunomodulators and tissue markers. One property of lectins is their ability to combine with sugars and glycoconjugates. Lectins have been shown to have a role in the prevention of cancers, in the activation of immune system, and in antimicrobial and insecticidal mechanisms. In addition, they may be used in the control of obesity [42].

Hypocholesterolemic, glucose, and blood pressure-lowering actions have been reported for both proteins and peptides by in vitro and clinical studies. Conglutin γ from lupin has been demonstrated to bind insulin (K_d_ = 9 × 10^−5^ M), thus controlling glucose plasma levels [43].

High biological properties of protein extracts of local varieties of *Phaseolus vulgaris* L., such as antiradical, anti α-amylase, and angiotensin converting enzyme-inhibitory activity [44], as well as influence on intestinal permeability, have recently been described [45].

## 4. Structure–Digestibility Relationship of Legume Proteins

The structural properties of legume proteins, by imparting high stability during gastrointestinal digestion, have been reported to play an important role in their in vivo bioactivity and release of bioactive sequences [46,47].

Major structural properties of legume proteins that have been described with nutraceutical activity are reported in Table 2.

The anticarcinogenic effect of BBI towards colon cancer has been related to the native conformation of the inhibitory domain, the inhibitor being found intact in several organs (liver, lung) after ingestion [40]. In particular, trypsin and chymotrypsin inhibitors of the BBI class of both soybean and pea seeds have been found to present anticarcinogenic effects: soybean inhibitors are active toward colon, liver, lung, esophagus, and breast cancers, while pea inhibitors present anti-proliferative activity toward colon cancer [41,42].

Preservation of the conformation of conglutin γ has resulted in being a prerequisite for insulin binding and hypoglycemic activity of the protein, tested in a rat model [43]. Similar properties have been found for the basic 7S globulin, a protein with 64% identity to conglutin γ isolated from soybean seed and built up by two disulfide-bridged subunits of 27 and 16 kDa [43].

Trypsin inhibitors and lectins have been shown to be internalized by the small intestinal villi of rat [48]. These proteins are very stable during processing and gastrointestinal digestion [36].

In addition to stability conferred by disulfide bonds, hydrophobicity is known to affect the physicochemical properties (hydration, gelation, emulsification, foaming, adhesion) of plant proteins, with a consequence on both absorption and nutritional properties. Soybean protein extract showed an average hydrophobicity of 6.44 kJ per residue and control of bitterness of soy hydrolysates in relation to hydrophobicity was successful to increase their functionality.

In oligomeric storage proteins of legume seeds, stabilization conferred by hydrophobic patches between monomers is likely to decrease susceptibility to proteolysis, especially after technological processing [10]. As a consequence, essential amino acids and bioactive peptides may be imprisoned inside stable complexes that are no longer digested [36].

Other adverse effects may include immunological reactions promoted by soluble and stable protein complexes [48]. Major allergens that have been found to be responsible for sensitization are α- and β-conglutins from lupin. Peanut, lentil, and soybean allergens have also been identified. The major lentil allergen is Len c1 (a 48 kDa vicilin), while 33 proteins from soybean (7–71 kDa) have been found to be allergenic. Stability of these proteins during gastrointestinal digestion has been reported as a major cause for their allergenicity [36,49].

**Figure 1:**
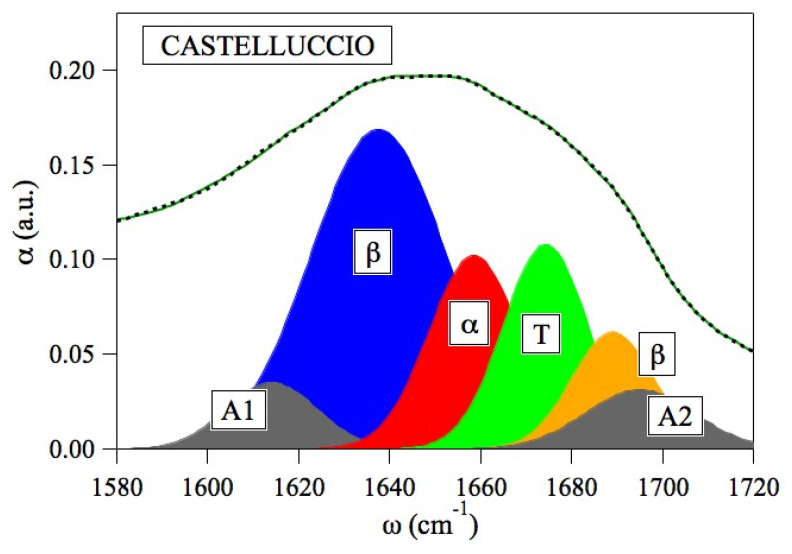
Analysis of proteins of Castelluccio lentil PGI (protected geographical indication) by FTIR. The amide I band was deconvolved by gaussian contributes. A1: intermolecular aggregates; β: β-sheet; α: α-helix; T: turns, A2: β-aggregates. (Carbonaro and Nucara, personal communication).

Fourier transform infrared spectroscopy (FTIR) has recently been employed to analyze the relationship between structure and bioavailability of food protein by examination of the amide I of the spectrum [46]. FTIR has demonstrated that the secondary structure of several plant proteins, such as legume proteins, is dominated by contributions from β-sheet conformation and, to this respect, it markedly differs from that of animal proteins, characterized by α-helix structure (Figure 1).

**Figure 2:**
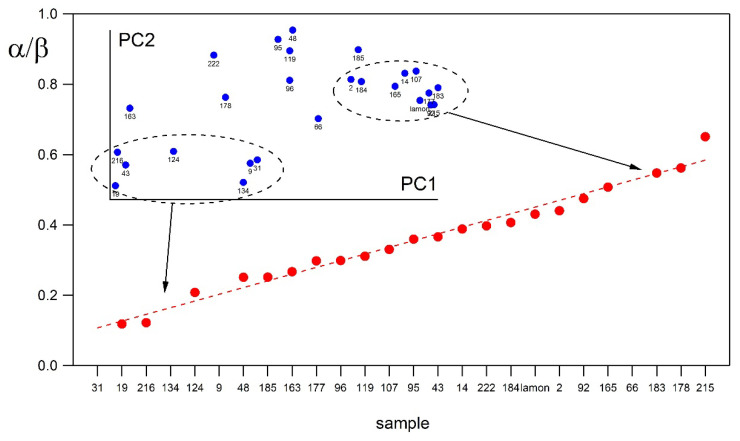
Ratio between percentage of α-helix and β-sheet secondary structures for common bean varieties (red points and dashed line). Dashed line is a guide for the eyes. In the inset the score-plot of PC1 and PC2 obtained from a PCA analysis on fit results is reported. (Carbonaro and Nucara, personal communication).

Legume proteins presented quite a lower α-helix to β-sheet ratio than cereal proteins (0.47 and 1.1, respectively) [46]. A different α-helix to β-sheet ratio was found in a recent study on several varieties of *P. vulgaris* coming from different countries, with cannellini and borlotti varieties showing the highest values (form 0.47 to 0.56) (Figure 2).

Moreover, β-sheet content of legume proteins has been found to account for the formation of stable intermolecular complexes upon thermal treatment. A high correlation between β-sheet content and protein digestibility has been found for food proteins and for both native and heated legume proteins [46]. Hydrophobic amino acids (alanine, valine, methionine, isoleucine, phenylalanine), together with cysteine, have been found in the small intestinal content of rats fed with legume proteins, further supporting the role of the structural properties of these proteins on the overall nutritional quality [14].

**Figure 3:**
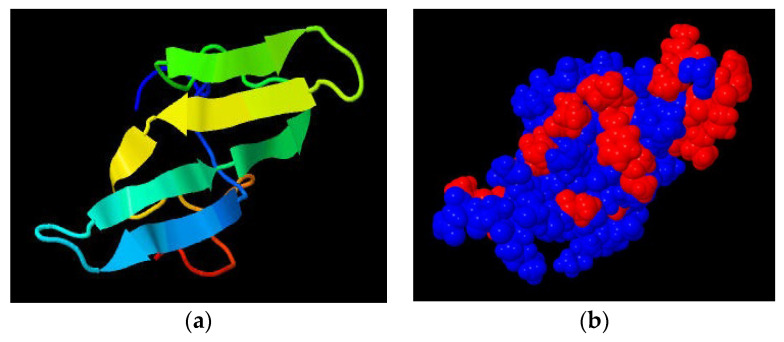
3D structure (**a**) and surface properties (**b**) of soybean Bowman–Birk inhibitor as rendered by Jmol software using the coordinates available in the Brookhaven Protein Data Bank (http://www.rcsb.org/pdb) (accessed on 15 December 2021). Surface color mapping: blue for hydrophilic, red for hydrophobic regions.

3D structure and surface features of the Bowman–Birk inhibitor are presented in Figure 3a and Figure 3b, respectively. It is evident that this protein is dominated by β-sheet conformation and by large hydrophobic areas on the surface. Stability is conferred by seven disulfide bridges in a small molecular weight protein (8 kDa). Other legume seed proteins (storage globulins, α-amylase inhibitors) have been shown to present similar conformational attributes. These features limit digestibility and digestion rate of the protein in the gastrointestinal tract [46,50].

Exogenous factors may also adversely affect digestibility of legume proteins: these include interaction with other compounds such as carbohydrates, tannins, phytates, lipid, trypsin inhibitors, and lectins [14,36].

Structural properties of legume proteins are likely to also have a role on bioavailability of some micronutrients, such as Fe.

Legume (lentil, chickpea, and pea) seed ferritin concentrates (30–45 mg Fe/100 g) have been investigated for the relationship between structure and resistance to digestion [51]. A correlation between concentration in Asx + Glx of the different legume proteins and iron content was found.

Most of the iron was released by pepsin digestion. Therefore, these iron-rich protein extracts are candidates in the production of functional foods to be used in place of inorganic iron against iron deficiency anemia. The two ferritin polypeptides showed a loose structure, as evidenced by intrinsic fluorescence spectroscopy. This property likely enabled protein degradation and iron release at low pH in the stomach [51].

In another study, determination of Cu, Fe, and protein absorption in the small intestine of rat has been carried out in single-dose experiments [52]. After thermal treatment of legume seeds, most (about 80%) of compounds was extracted in the insoluble fraction. Absorption of proteins, Cu, and Fe in this fraction was low, indicating that insolubilization negatively influences protein, Cu, and Fe absorption from legumes. Increased hydrophobicity of legume proteins after thermal treatment induced protein aggregation and precipitation into insoluble complexes.

These findings suggest that the structure of legume proteins is likely to affect bioavailability not only of essential amino acids, but also that of some micronutrients, such as Cu and Fe.

## 5. Structural Traits of Bioactive Peptides

Similarly to bioactive peptides from animal proteins (milk, meat), those derived from legume proteins are characterized by: (i) a short length (2–20 amino acids); (ii) proline, lysine, arginine, and hydrophobic amino acids; (iii) low susceptibility to digestion [53,54].

Proteolytic digestion of soybean 7S protein produces a pentapeptide (Leu–Leu–Pro–His–His) that has shown antioxidant activity. An Arg–Gly–Asp tripeptide has been found to be the adhesion region of soy lunasin to the cells, adhesion being a prerequisite for its anticarcinogenic properties [55].

Lunasin is a peptide with 44 amino acids and a high content in Asp, extracted from soybean 2S albumins and then isolated from cereal (wheat, barley, rye) proteins [56]. Besides lunasin, Val–Pro–Tyr and γ-glutamyl peptides from soybean also have anti-inflammatory properties [57]. Hydrophobic peptides from soybean also present anticarcinogenic properties [58].

The antioxidant activity of 28 short-chain peptides attributed to Leu–Leu–Pro–His–His have been examined: the tripeptide Pro–His–His has been shown to be active as metal chelator or radical scavenger, increasing the antioxidant properties of soy protein hydrolysate [59].

Recently, several peptides with antioxidant properties and a high amino acid score have been isolated after hydrolysis of legume proteins [60].

It has also been shown that proteins with low content of Met–Gly and Lys–Arg, i.e., soy and fish proteins, lower cholesterol level [61].

Besides specific residues, charge properties, hydrogen bonding and hydrophobicity are believed to influence both susceptibility to proteolysis and peptide absorption, besides the physiological functions of peptides.

Modern in silico techniques, such as quantitative structure–activity relationship (QSAR) models, consisting in analysis of homology similarity, are available for the screening of the origin of bioactive peptides [62,63,64]. These approaches have allowed the discovery of bioactive peptides, based on their sequence similarity.

Results from bioinformatic predictions have indicated that fragments with probability to be produced are hydrophilic and, therefore, are present at the external surface of the protein. These regions contain a high percentage of random coil (46%) and low amounts of β-sheet (17%) [65].

Bioinformatic tools, based on different algorithms, may help in predicting enzymatic hydrolysis of proteins to account for proteolytic process designs.

## 6. Conclusions

The structural properties of legume proteins, as also evidenced by FT-IR analysis, by imparting high stability during processing and gastrointestinal digestion, are likely to affect their bioactivity and production of bioactive peptides.

Further knowledge of the relationships between structure and bioactivity of protein and peptides from legume seeds is required to optimize their use as nutraceuticals, to increase peptide production, and to improve bioavailability of bioactive sequences. Such information may also be useful in planning strategies for eliminating the risk of adverse reactions, such as allergenicity, consequent to consumption of legumes for sensitive population groups, another aspect that has partially been related to high protein stability in the gastrointestinal tract.

## Figures and Tables

**Table 1:** Legume proteins and peptides with nutraceutical properties (modified from reference [36]).

Precursor	Name/Sequence	Nutraceutical Activity
Soy trypsin/chymotrypsin inhibitor	Kunitz/Bowman Birk inhibitor	Anticancer, anti-inflammatory, weight control
Soy amylase inhibitors	α-Amylase inhibitor	Antiobesity, antidiabetic, anticancer
Jack bean haemagglutinins	Concanavalin A, Lectins	Anticancer, immunostimolant
Bean, soy storage 7S globulins	Phaseolin, conglycinin, 7S protein α’ chain	Hypocholesterolemic
Soy storage 11S globulins	Hydrophobic peptides	ACE-inhibitory
Soy 2S albumins	Lunasin	Immunostimolant, anticancer, ipotensive
Lupin/soy conglutin γ	Conglutin	Hypoglycemic, hypocholesterolemic
Soy proteins	YPFVV, LPYPR, IAVPGEVA	ACE-inhibitory, antioxidant, opioid agonist
Fermented soybean	LVQGS	Antihypertensive

**Table 2:** Major structural properties of legume proteins with nutraceutical activity (modified from reference [36]).

Protein	MW (KDa)	Structure Type ^a^	α-Helix (%)	β-Sheet (%)	N° of SS
Kunitz trypsin inhibitor ^b^	21.5	Globular, monomeric	6	40–60	2
Bowman–Birk inhibitor ^b^	8	Globular, monomeric	0	60	7
α-Amylase inhibitors ^c^	12–60	Globular, monomeric/dimeric/tetrameric	15–30	25–60	2–5
Concanavalin A ^d^	110	Globular, tetrameric	0	47	0
Phaseolin ^e^	150	Globular, trimeric	16	37	0
Glycinin ^b^	340	Globular, oligomeric	15	36	22
Conglycinin ^b^	200	Globular, oligomeric	15	31	2
Conglutin γ ^f^	200	Globular, tetrameric	15	35	24

^a^ In phosphate-buffered saline, pH 7.0; ^b^ From soybean; ^c^ From cereals and legumes; ^d^ From jack bean; ^e^ From common bean; ^f^ From lupin.
